# Detection of Synaptic Proteins in Microglia by Flow Cytometry

**DOI:** 10.3389/fnmol.2020.00149

**Published:** 2020-09-29

**Authors:** Simone Brioschi, Paolo d’Errico, Lukas S. Amann, Hana Janova, Sonja M. Wojcik, Melanie Meyer-Luehmann, Lawrence Rajendran, Peter Wieghofer, Rosa C. Paolicelli, Knut Biber

**Affiliations:** ^1^Faculty of Biology, University of Freiburg, Freiburg, Germany; ^2^Department of Psychiatry, University of Freiburg Medical Center, Freiburg, Germany; ^3^Department of Neurology, University of Freiburg Medical Center, Freiburg, Germany; ^4^Institute of Neuropathology, University of Freiburg Medical Center, Freiburg, Germany; ^5^Department of Clinical Neuroscience, Max Planck Institute of Experimental Medicine, Göttingen, Germany; ^6^Department of Molecular Neurobiology, Max Planck Institute of Experimental Medicine, Göttingen, Germany; ^7^Institute for Regenerative Medicine, University of Zürich, Zürich, Switzerland; ^8^Institute of Anatomy, Leipzig University, Leipzig, Germany; ^9^Department of Biomedical Sciences, University of Lausanne, Lausanne, Switzerland

**Keywords:** microglia, synaptic pruning, VGLUT1, 5xFAD model, TDP-43 conditional knock-out

## Abstract

A growing body of evidence indicates that microglia actively remove synapses *in vivo*, thereby playing a key role in synaptic refinement and modulation of brain connectivity. This phenomenon was mainly investigated in immunofluorescence staining and confocal microscopy. However, a quantification of synaptic material in microglia using these techniques is extremely time-consuming and labor-intensive. To address this issue, we aimed to quantify synaptic proteins in microglia using flow cytometry. With this approach, we first showed that microglia from the healthy adult mouse brain contain a detectable level of VGLUT1 protein. Next, we found more than two-fold increased VGLUT1 immunoreactivity in microglia from the developing brain (P15) as compared to adult microglia. These data indicate that microglia-mediated synaptic pruning mostly occurs during the brain developmental period. We then quantified the VGLUT1 staining in microglia in two transgenic models characterized by pathological microglia-mediated synaptic pruning. In the 5xFAD mouse model of Alzheimer’s disease (AD) microglia exhibited a significant increase in VGLUT1 immunoreactivity before the onset of amyloid pathology. Moreover, conditional deletion of TDP-43 in microglia, which causes a hyper-phagocytic phenotype associated with synaptic loss, also resulted in increased VGLUT1 immunoreactivity within microglia. This work provides a quantitative assessment of synaptic proteins in microglia, under homeostasis, and in mouse models of disease.

## Introduction

Microglia originate through primitive hematopoiesis in the yolk sac and colonize the brain rudiment during embryonic development (Ginhoux et al., 2010; Schulz et al., 2012; Kierdorf et al., 2013). During the perinatal period, microglia acquire a highly ramified morphology and appear evenly distributed throughout the brain parenchyma (Ginhoux and Prinz, 2015). Microglia *in vivo* are extremely motile cells exhibiting continuous extension and retraction of their finely ramified processes, thus performing a ceaseless immunological surveillance of the surrounding environment (Davalos et al., 2005; Nimmerjahn et al., 2005; Hanisch and Kettenmann, 2007). Moreover, microglia support the formation and consolidation of neural circuits in the developing brain (Parkhurst et al., 2013; Ueno et al., 2013; Squarzoni et al., 2014; Zhan et al., 2014; Miyamoto et al., 2016). Under homeostasis, microglia constantly interact with synaptic inputs (Wake et al., 2009; Tremblay et al., 2010), thus mediating a fine-tuning of the synaptic activity (Kettenmann et al., 2013). Importantly, during brain development microglia enact displacement/removal of synaptic inputs, thus actively contributing to synaptic pruning (Stevens et al., 2007; Paolicelli et al., 2011; Schafer et al., 2012; Filipello et al., 2018; Lehrman et al., 2018). Very recently, abnormal synaptic refinement by microglia was also reported in various mouse models of neurological disorders (Hong et al., 2016; Lui et al., 2016; Schafer et al., 2016; Vasek et al., 2016; Paolicelli et al., 2017; Shi et al., 2017; Di Liberto et al., 2018). A full elucidation of this mechanism will provide a deeper understanding of the dynamics of brain development, as well as of neurodevelopmental and neurodegenerative disorders (Neniskyte and Gross, 2017; Salter and Stevens, 2017). At present, further progress in this field of research is hampered by the lack of standardized techniques allowing an accurate estimation of synaptic material in microglia. To our knowledge, all the available data are essentially generated with microscopy-based approaches, such as confocal imaging and electron microscopy (EM; Schafer et al., 2012; Bisht et al., 2016; Sipe et al., 2016). Modern microscopy provides a qualitative appraisal of synaptic proteins inside of microglia, yet for a fast and unbiased quantification, microscopy-based techniques have some drawbacks. In particular, the spatial resolution of confocal microscopy may be insufficient to resolve microglial and synaptic structures when they are less than few hundreds of nanometers apart from each other (Weinhard et al., 2018). Given these technical limitations, the development of alternative approaches is currently in high demand (Sierra et al., 2013). In the present work, we used flow cytometry intracellular staining to quantify VGLUT1, which is a marker for glutamatergic synapses, in microglia acutely isolated from different mouse models. In flow cytometry, VGLUT1 immunoreactivity was detected in microglia, but not in other extra-parenchymal CD45^hi^ brain macrophages. We then provided evidence that VGLUT1 is localized inside microglial cells and is not due merely to adhesion of synaptic proteins on the cell surface. Interestingly, significantly increased VGLUT1 immunoreactivity was found in microglia from juvenile mice (post-natal day 15) than in microglia from adult mice (12-week-old), supporting the hypothesis that synaptic pruning occurs primarily in brain development. Similarly, microglia from 2-month-old 5xFAD mice exhibited higher VGLUT1 staining compared to wild-type controls, indicating increased pruning of glutamatergic synapses at the early stages of amyloid pathology. Last, augmented VGLUT1 was also found in TDP43-deficient microglia, which was previously shown to exhibit an hyper-phagocytic phenotype (Paolicelli et al., 2017). By providing a quantitative assessment, our data consolidate previous evidence concerning the uptake of synaptic material by microglia in various mouse models of brain disease.

## Materials and Methods

### Animals and Ethics

All animal experiments were performed with the permission of the local authorities of the Regional Council of Freiburg (Regierungspräsidium) and the animal welfare committee of the University of Freiburg, and with approval of the animal care and use committees of the Swiss Cantonal Veterinary Office. All mice used here (including wild-type, *Cx3cr1^GFP/+^*, *Vglut1^−/−^*, *5xTgFAD* and *Cx3cr1^CreERT2/+^;Tardbp^flox/flox^*) were bred on a C57BL/6J background and maintained under specific pathogen-free conditions, in a temperature- and humidity-controlled facility with a 12 h light-dark cycle. Food and water were available *ad libitum*. To minimize gender-dependent heterogeneity of amyloid pathology in *5xTgFAD* mice, males only were used. *5xTgFAD* and wild-type control mice were analyzed at the age of 2 months, therefore prior to the amyloid-plaques formation. For all the other experiments both genders were used, and mice were analyzed at the age of either 2 months or post-natal day 15 (P15). The generation and characterization of the *Vglut1* knock-out line used here were previously described (Wojcik et al., 2004). *Vglut1* knock-out mice and control wild-type littermates were analyzed at the age of P15–P17 because of the premature death owing to *Vglut1*-deficiency. To induce the recombination of the *Tardbp* floxed allele in microglia, *Cx3cr1^CreERT2/+^;Tardbp^flox/flox^* mice (TDP-43 conditional knockout) underwent 5 i.p. injections 24 h apart of corn oil-dissolved Tamoxifen (1.5 mg/mouse/day; Sigma–Aldrich, Buchs, Switzerland). Tamoxifen treatment began at P30 and mice were sacrificed at P90. Importantly, the insertion of the *Cre* transgene downstream of the *Cx3cr1* promoter disrupts expression of the endogenous *Cx3cr1* gene. Given the risk of experimental bias due to the monoallelic expression of *Cx3cr1*, we used *Cx3cr1^CreERT2/+^;Tardbp^+/+^* littermates as the wild-type control. The same tamoxifen treatment was applied on both *Cx3cr1^CreERT2/+^;Tardbp^flox/flox^* and *Cx3cr1^CreERT2/+^;Tardbp^+/+^* mice to normalize possible drug-related effects.

### Immunofluorescence

Mice were deeply anesthetized through intraperitoneal (i.p.) injection of ketamine hydrochloride (Ketavet, Pfizer; dosage 300 mg/kg body weight) and xylazine (Rompun, Bayer HealthCare; dosage 30 mg/kg body weight). Mice were then intracardially perfused with ice-cold PBS. Dissected brains were fixed in 4% PFA (paraformaldehyde) overnight at 4°C and subsequently cryoprotected in 30% sucrose solution for 48 h at 4°C. Frozen whole brains were cut into 60 μm thick coronal sections. Free-floating sections were pre-incubated for 2 h at room temperature with blocking solution (PBS + 5% horse serum and 0.5% Triton X-100) and subsequently incubated 48 h at 4°C with the primary antibody solution (PBS + 1% horse serum and 0.5% Triton X-100). Sections were then washed and incubated overnight at 4°C with secondary (fluorochrome-conjugated) antibody and DAPI for nuclear staining (1:4,000, Sigma). After washing sections were mounted with ProLong Diamond Antifade Mounting Medium (Thermo Fisher Scientific).

**Table 1:** 

**Antibody**	**Product**	**Clone**	**Host**	**Dilution**
Anti-Iba1	Wako Chemicals, 019-19741	Polyclonal	Rabbit	1:500
Anti-CD68	Bio-Rad, MCA1957GA	FA-11	Rat	1:200
Anti-VGLUT1	Merck Millipore, MAB5502	3C10.2	Mouse	1:500
Anti-NeuN Alexa-488	Merck Millipore, MAB377	A60	Mouse	1:1,000
Anti-Amyloid-Beta	Biolegend, Sig-39320	6E10	Mouse	1:1,000
Anti-Mouse Alexa-488	Life Technologies, A21202	Polyclonal	Donkey	1:1,000
Anti-Rat Alexa-488	Abcam, ab150153	Polyclonal	Donkey	1:1,000
Anti-Mouse Alexa-555	Thermo Fisher Scientific, A-21424	Polyclonal	Goat	1:1,000
Anti-Mouse Alexa-568	Life Technologies, A11004	Polyclonal	Goat	1:1,000
Anti-Rabbit Alexa-647	Life Technologies, A31573	Polyclonal	Donkey	1:1,000
**Antibody**	**Product**	**Clone**	**Host**	**Dilution**
Anti-CD11b BV421	Biolegend, 101235	M1/70	Rat	1:200
Anti-CD45 APC	eBioscience, 17-0451-82	30-F11	Rat	1:200
Anti-VGLUT1 PE	Merck Millipore, FCMAB335PE	3C10.2	Mouse	0.5 μg/test
Anti-VGLUT1 Oyser550	Synaptic Systems, 135303C3	Polyclonal	Rabbit	0.5 μg/test
Anti-Synaptophysin	Merck Millipore, MAB5258-20UG	SY38	Mouse	0.5 μg/test
IgG1 K Isotype Control PE	eBioscience, 12-4714-42	P3.6.2.8.1	Mouse	0.5 μg/test
Anti-HA Tag Oyser550	Synaptic Systems, 245003C3	Polyclonal	Rabbit	0.5 μg/test

Amyloid-β immunostaining was performed on 25 μm thick coronal sections. Free-floating sections were incubated overnight with the anti-Aβ 6E10 primary antibody (1:1,000, Biolegend). Staining with the secondary (fluorochrome-conjugated) antibody was performed for 2 h at room temperature. Dense-core plaques were stained with Thiazine-red (2 μM, Sigma) for 5 min at room temperature. Sections were then counterstained with DAPI (1:10,000, Sigma) and mounted with fluorescence mounting medium (DAKO).

### Confocal Imaging

Immunostained 60 μm thick brain cryosections were analyzed by confocal laser scanning ZEISS LSM 510 META microscope. z-stack images were obtained with either 25×/0.8 NA or 63×/1.4 NA oil-immersion objectives (Zeiss). Microscope settings: 1.6 μs pixel dwell, resolution 2,048 × 2,048 pixels, averaging number 4, *z*-step 0.5 μm, unidirectional acquisition mode, color depth 12 bit. Confocal images were initially deconvolved with Huygens Professional software^1^ using default parameter settings and subsequently analyzed using IMARIS software version 7.5.3^2^. Amyloid-β covered area was calculated with ImageJ using the default auto-threshold. Manual threshold adjustments were applied if necessary. Quantification of the VGLUT1+ synaptic inputs was carried out using the Imaris spot-detection tool. The automatic detection algorithm was set to identify all VGLUT1+ spots with a diameter ≥1 μm. For thresholding, 10% of dimmest spots were excluded.

### STED Microscopy

STED images were acquired utilizing STEDYCON technology (Abberior Instruments GmbH) installed on a ZEISS LSM 510 META confocal microscope carrying a 100×1.45 NA oil-immersion objectives (Zeiss). Immunofluorescent labeling for Iba1 and VGLUT1 was carried out as described above. Anti-mouse-Star580 and anti-rabbit-StarRED (diluted 1:100) were used as secondary (fluorochrome-conjugated) antibodies. Secondary antibodies were a kind gift from Dr. Janina Hanne of Abberior Instruments GmbH^3^. Raw images were deconvolved with Huygens Professional software and analyzed in IMARIS 7.5.3 software.

### Flow Cytometry

After intracardiac perfusion with ice-cold PBS, brains were harvested and slowly homogenized with a tissue potter and filtered through a 70 μm strainer. Myelin was then removed by centrifugation on 30% percoll gradient. Brain pellets were sequentially stained with live/dead staining and for the microglial surface markers CD11b and CD45. Subsequently, stained samples were fixed and permeabilized using the BD Cytofix/Cytoperm kit. Eventually, we performed intracellular staining for the desired synaptic marker. Samples were acquired on BD FACS Canto II (BD Bioscience) flow cytometer. Raw data were analyzed with FlowJo v10 (BD Bioscience). See the protocol for a more detailed description of the method.

### Statistics

Graphs and statistics were produced using the Graph-Pad Prism 5 software package. Statistical difference between two groups was determined by either a two-tailed unpaired Student’s *t*-test or two-tailed Mann–Whitney *U*-test. To test for normal distribution, the Kolmogorov–Smirnov test was used. When the data passed the normality test (α = 0.05), the *p*-value was determined with the unpaired Student’s *t*-test. When the data did not pass the normality test (α = 0.05), or the *N* size was too small, the *p*-value was determined with the Mann–Whitney *U*-test. The exact *p*-values were reported. Data are shown as the mean value ± SEM.

## Results

### VGLUT1 Inclusions Can Be Found in Adult Hippocampal Microglia Under Homeostasis

**Figure 1:**
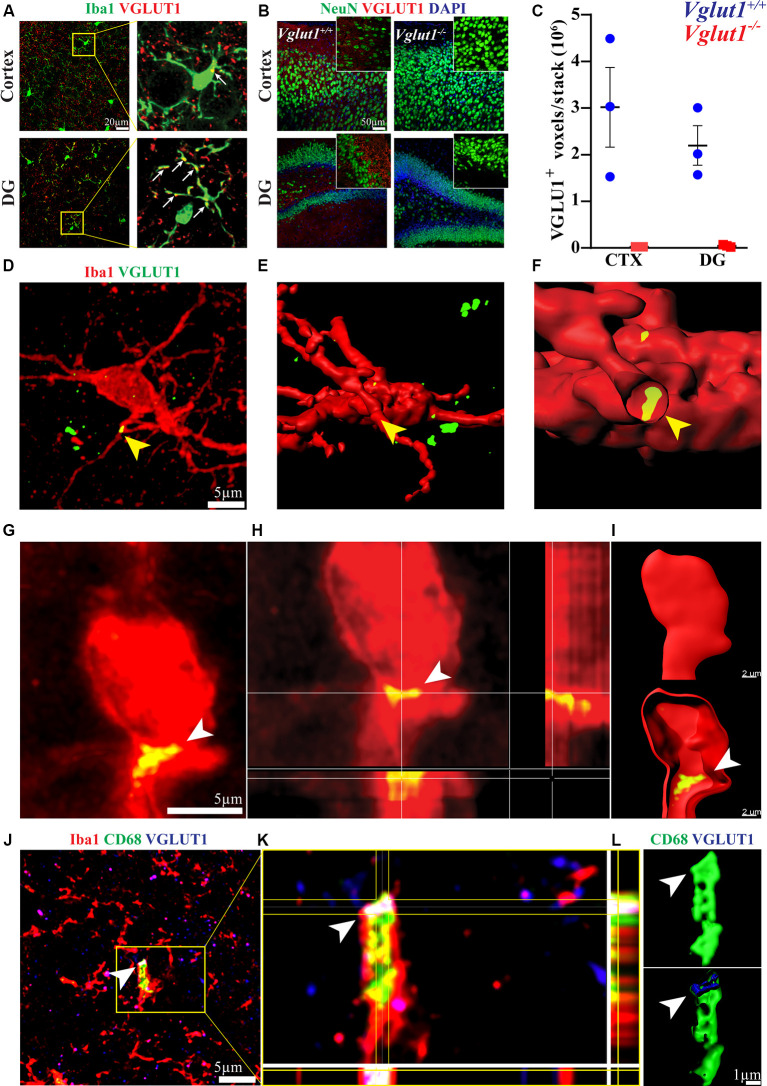
VGLUT1 inclusions can be found in adult hippocampal microglia under homeostasis. **(A)** In immunohistochemistry, frequent overlaps between Iba1 and VGLUT1 signals can be detected in the brain cortex and dentate gyrus (DG). **(B)** Anti-VGLUT1 staining in cortex and dentate gyrus (DG) from both *Vglut1^+/+^* (wild-type) and *Vglut1*^−/−^ (knock-out) littermates. **(C)** Quantification of the VGLUT1-positive voxels per stack in cortex (CTX) and dentate gyrus (DG) of both *Vglut1^+/+^* and *Vglut1*^−/−^ mice (*N* = 3 mice per group, two sections per mouse; data from a single experiment). **(D)** Microglial cell at the CA1 hippocampus exhibiting a VGLUT1 inclusion overlapping with a process (yellow arrowhead). **(E)** 3D cell reconstruction of the same microglial cell displayed in panel **(D)**. **(F)** Application of a clipping surface on the 3D reconstruction discloses the presence of a VGLUT1 inclusion within the microglial process (yellow arrowhead). **(G)** STED image of a microglial cell in the CA1 exhibiting a VGLUT1 inclusion (white arrowhead). **(H)** Orthogonal projection of the imaged in panel **(J)** showing the intracellular localization of the VGLUT1 staining. **(I)** 3D reconstruction of the cell in panel **(J)** showing the intracellular localization of the VGLUT1 staining. **(J)** A confocal image of a microglial cell body exhibiting VGLUT1 inclusion overlapping with a CD68^+^ phagosome (scale bar 5 μm). **(K)** Orthogonal projection of the inset area. Note the colocalization between VGLUT1 and CD68 staining (white spot in the centre of the crosshair). **(L)** 3D reconstruction of both CD68 (green) and VGLUT1 (blue) signals showing that the VGLUT1 inclusion is perfectly encased within the microglial phagosomes (white arrowhead).

In the adult mouse brain, microglia constantly engage contacts with the surrounding neuronal dendrites (Supplementary Figure S1A) and this phenomenon is apparently driven by neuronal activity (Wake et al., 2009; Tremblay et al., 2010; Stowell et al., 2019). We analyzed the brain of 12-week-old mice by confocal imaging, looking for evidence of synaptic engulfment in microglia. Immunostaining for Iba1 (a marker for microglia) and VGLUT1 (a marker for glutamatergic pre-synaptic inputs) showed that microglial processes often overlap with glutamatergic synapses in both cortex and dentate gyrus (Figure 1A). To exclude possible bias due to a non-specific staining, we tested the used anti-VGLUT1 antibody on *Vglut1* knock-out (*Vglut1*^−/−^) mice (Figure 1B, Supplementary Figure S1B). Confocal imaging in the cortex and dentate gyrus shows that the used antibody did not produce detectable staining in *Vglut1*^−/−^ mice (Figure 1C). Moreover, VGLUT1 staining in Thy1-EGFP mice showed that VGLUT1 synapses are localized in proximity to the neuronal dendrites (Supplementary Figure S1C). These data together indicate that the used anti-VGLUT1 antibody was specific for glutamatergic synapses. We, therefore, decided to focus on VGLUT1 as a synaptic marker of interest.

In the hippocampus of 12-week-old mice, we could occasionally observe the presence of VGLUT1 immunoreactivity localized within the microglial cytoplasm (Figure 1D). Intracellular localization of VGLUT1 staining was confirmed by 3D cell reconstruction (Figures 1E,F). Such inclusions were found in both microglial processes (Supplementary Figure S1D) and cell bodies (Supplementary Figure S1E). The presence of VGLUT1 staining inside microglia was further confirmed by STED microscopy (Figures 1G,H) and 3D cell reconstruction (Figure 1I). To determine whether VGLUT1 inclusions in microglia stemmed from phagocytosis of synapses we performed triple staining for Iba1, VGLUT1, and CD68 (a marker for phagolysosomes in macrophages). Our images show that VGLUT1 inclusions in microglia colocalized with phagosomes (Figures 1J,K). Moreover, 3D reconstruction of the CD68-positive surface confirmed that the VGLUT1 inclusions were located within the phagosomes (Figure 1L), indicating that glutamatergic pre-synaptic inputs were taken up by phagocytosis.

Although immunofluorescence staining may help appreciate the presence of glutamatergic synaptic proteins in microglia, we concluded that confocal imaging does not grant enough resolution power to reliably discriminate between the synapses that are truly engulfed and those that are simply juxtaposed to the microglial processes. Indeed, a recent work suggested caution to use confocal imaging to investigate synaptic pruning (Weinhard et al., 2018). As an alternative and complementary approach, we used flow cytometry to assess and quantify VGLUT1 immunoreactivity in microglia.

### Flow Cytometry Allows for Relative Quantification of VGLUT1 Immunoreactivity in Microglia From the Adult Healthy Brain

**Figure 2:**
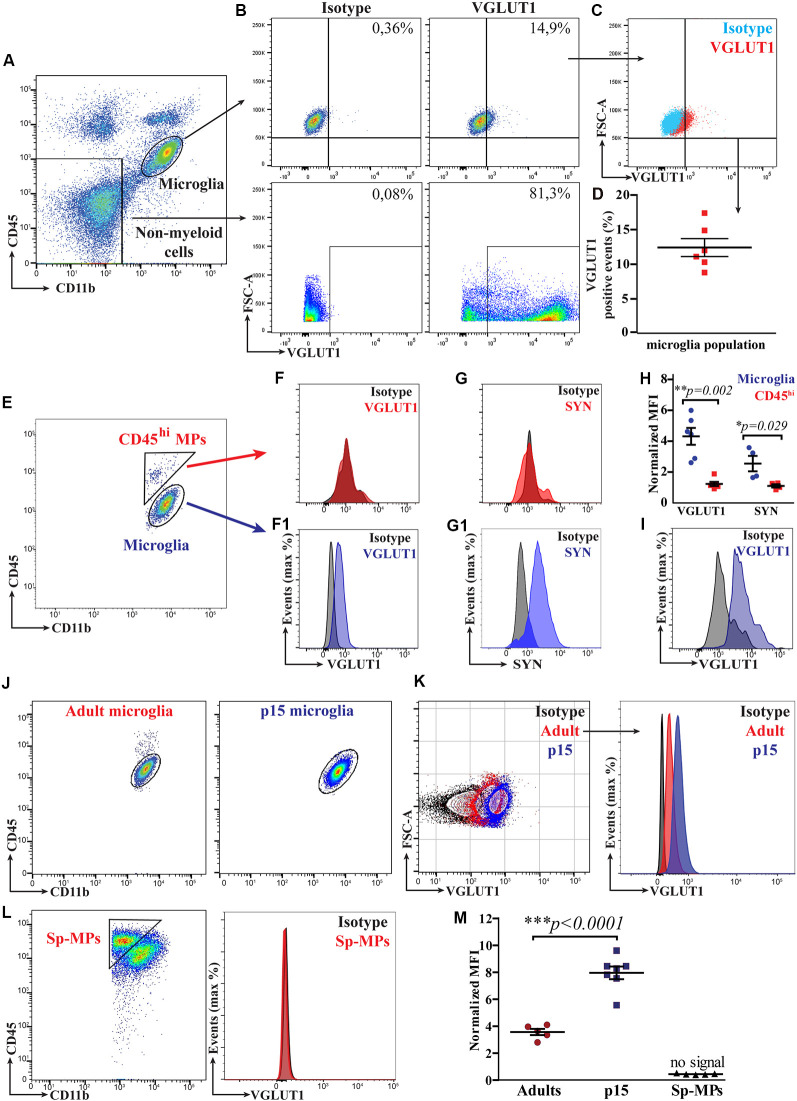
Flow cytometry allows for a relative quantification of VGLUT1 immunoreactivity in microglia from both the adult and the developing mouse brain. **(A)** FACS plot displaying the gates for both microglia (CD11b^+^CD45^lo^ population) and brain’s non-myeloid cells (CD11b^−^CD45^−^ population). **(B)** FACS plot showing VGLUT1 immunoreactivity in both microglia and brain’s non-myeloid cells. **(C)** Microglia from VGLUT1-stained samples exhibit a positive staining, as compared to isotype control. **(D)** Percentage of the VGLUT1-positive microglia, fluorescence of the isotype control is used as baseline (*N* = 6 mice, pooled data from two independent experiments). **(E)** FACS plot displaying both microglia (CD11b^+^CD45^lo^ population) and CD45hi MPs (CD11b^+^CD45^hi^ population). **(F)** Fluorescence peak of the VGLUT1 staining in CD45^hi^ MPs. **(F1)** Fluorescence peak of the VGLUT1 staining in microglia (anti-VGLUT1-PE, Merck Millipore FCMAB335PE, clone 3C10.2). **(G)** Fluorescence peak of the Synaptophysin staining in CD45^hi^ MPs. **(G1)** Fluorescence peak of the Synaptophysin staining in microglia. **(H)** n-MEAN-FLUORESCENCE-INTENSITY (MFI) for VGLUT1 and Synaptophysin in both microglia and CD45^hi^ MPs (VGLUT1 *N* = 6 mice per group, pooled data from two independent experiments; Mann–Whitney *U*-test; **p = 0.002. Synaptophysin *N* = 4 mice per group, data from a single experiment; Mann–Whitney *U*-test unpaired; *p = 0.029). **(I)** Fluorescence peak of the VGLUT1 staining in microglia using an alternative antibody clone conjugated with a different dye (anti-VGLUT1-Oyster550, Synaptic Systems 135303C3, polyclonal). **(J)** FACS plot displaying microglia from either adult (12-week-old) or P15 mice. **(K)** Comparison of the VGLUT1 immunoreactivity between adult and P15 microglia. **(L)** VGLUT1 staining was not detectable in splenic macrophages (Sp-MPs). **(M)** VGLUT1 n-MFI in microglia from P15 and adult brain, Sp-MPs served as a negative control (adult *N* = 5 mice per group, P15 *N* = 7 mice per group, Sp-MPs *N* = 5 mice per group, data from a single experiment; unpaired *t*-test; ***p < 0.0001).

We first tested whether VGLUT1 staining could be reliably quantified in adult microglia by flow cytometry. To do so, we performed intracellular staining for VGLUT1 on brain homogenates from 12-week-old *Cx3cr1^GFP/+^* mice (gating strategy in Supplementary Figure S2). We then gated separately microglia (CD11b^+^CD45^lo^ population) and brain’s non-myeloid cells (CD11b^−^CD45^−^ population; Figure 2A). Microglia exhibited a weak yet detectable immunoreactivity for VGLUT1. By contrast, the brain’s non-myeloid cells were strongly positive for VGLUT1 (Figure 2B). Importantly, no VGLUT1 signal could be detected in the spleen homogenate, indicating that the used antibody did not cross-react with antigens outside the CNS (Supplementary Figure S3A). We then quantified VGLUT1 immunoreactivity in microglia by comparing VGLUT1-stained vs. isotype control samples (Figure 2C). Doing so, we could detect an average of 12.4% (±1.3%) VGLUT1-positive microglial cells in our samples (Figure 2D). However, this method of quantification is obviously biased by the manual gating. To provide an unbiased relative quantification of the VGLUT1 immunoreactivity (hereafter VGLUT1 IR) among different cell types, we normalized VGLUT1 Mean-Fluorescence-Intensity (MFI) to the isotype MFI in each population of interest (MFI VGLUT1: MFI Isotype, hereafter “n-MFI”). Using this formula, VGLUT1 nMFI was determined in both microglia and CD11b^+^CD45^hi^ macrophages (CD45^hi^ MPs) separately (Figure 2E). The CD45^hi^ MPs population comprises extra-parenchymal myeloid cells (mostly perivascular, meningeal, and choroid plexus macrophages) which are not in direct contact with the brain’s parenchyma (Kierdorf et al., 2019; Brioschi et al., 2020) and therefore should not be positive for VGLUT1. Indeed, microglia exhibited a VGLUT1 n-MFI = 4.3 ± 0.5 (meaning 4.3-fold increased MFI compared to the isotype), while no signal was detected in CD45^hi^ MPs (n-MFI = 1.2 ± 0.1; Figures 2F,F1). Similar results were obtained staining for the pre-synaptic marker Synaptophysin (SYN; n-MFI in microglia = 2.6 ± 0.5; n-MFI in CD45^hi^ MPs = 1.1 ± 0.1; Figures 2G,G1). Overall, these data indicate that different synaptic markers can be detected and quantified in microglia, but not in CD45^hi^ MPs, which served as an internal control (Figure 2H). To rule out possible bias due to unspecific staining, we performed an additional VGLUT1 staining using a different antibody clone, which yielded similar n-MFI values (n-MFI in microglia = 5.4 ± 0.7; Figure 2I). Additionally, we compared VGLUT1 IR between hippocampal and cortical microglia from 12-week-old mice. We found that microglia from hippocampus exhibit a slight, yet significant, increase of VGLUT1 n-MFI (5.5 ± 0.5) compared to microglia from cortex (3.9 ± 0.2; Supplementary Figure S3B). These data indicate that VGLUT1 IR in microglia varies depending on the brain region. In summary, this flow cytometry-based approach allowed for relative quantification of synaptic proteins in microglia from the adult healthy brain.

### VGLUT1 Immunoreactivity in Microglia Is Intracellular

To prove that the observed VGLUT1 IR in microglia was derived from a specific intracellular staining, we performed a control experiment treating brain homogenates with either FACS buffer or permeabilization buffer. In principle, without permeabilization, the antibody does not cross the cell membrane, resulting in a lack of intracellular staining. For this experiment, we prepared brain homogenates from *Cx3cr1^GFP/+^* mice, which were split equally into two separate tubes. Half of the sample was stained for VGLUT1 in FACS buffer, while the other half was stained using a permeabilization buffer. In flow cytometry, we gated both microglia (CX_3_CR1^+^CD11b^+^CD45^lo^) and non-microglial cells (CX_3_CR1^−^CD11b^−^CD45^−^) separately (Supplementary Figure S3C). In samples incubated with FACS buffer microglia did not exhibit detectable VGLUT1 IR, which was instead present using the permeabilization buffer (Supplementary Figure S3D). This difference was even more remarkable in the non-myeloid cells (Supplementary Figure S3E). Focusing on microglia, we measured a VGLUT1 n-MFI = 0.9 ± 0.1 using FACS buffer, and n-MFI = 6.8 ± 0.9 with the permeabilization buffer (Supplementary Figure S3F). This experiment showed that membrane permeabilization increased VGLUT1 IR in microglia of ~7-fold (Supplementary Figure S3G). To summarize, the permeabilization of the cell membrane was necessary for successful detection of VGLUT1 in microglia, hence, we concluded that the stained synaptic protein was located within intracellular compartments.

### P15 Microglia Exhibit Increased VGLUT1 Immunoreactivity as Compared to Adult Microglia

Early post-natal development is a critical period for brain maturation and synapse remodeling (Tau and Peterson, 2010). Recent literature has provided evidence that, in the early post-natal brain, microglia contribute to maturation of neural circuits by refining synaptic connections (Paolicelli et al., 2011; Schafer et al., 2012; Filipello et al., 2018). We hence aimed to quantify VGLUT1 staining in microglia from the developing brain (at postnatal day 15, P15) using flow cytometry (Figure 2J). We found that VGLUT1 IR was increased in microglia from P15 as compared to adult mice (Figure 2K). As expected, no VGLUT1 IR was detected in either splenic macrophages (Sp-MPs; Figure 2L) or CD45^hi^ MPs (not shown). According to our quantification, we measured 2.2-fold increased VGLUT1 n-MFI in P15 microglia (8.0 ± 0.5) compared to adult microglia (3.6 ± 0.2; Figure 2M). These data indicate that VGLUT1-positive microglia can be found in both the adult and the juvenile mouse brain. However, microglia during the post-natal brain development display a significantly higher VGLUT1 immunoreactivity, suggestive of an increased uptake of glutamatergic synapses in this period.

### Microglia Exhibit Increased VGLUT1 Immunoreactivity at Early Stages of Amyloid Pathology

**Figure 3:**
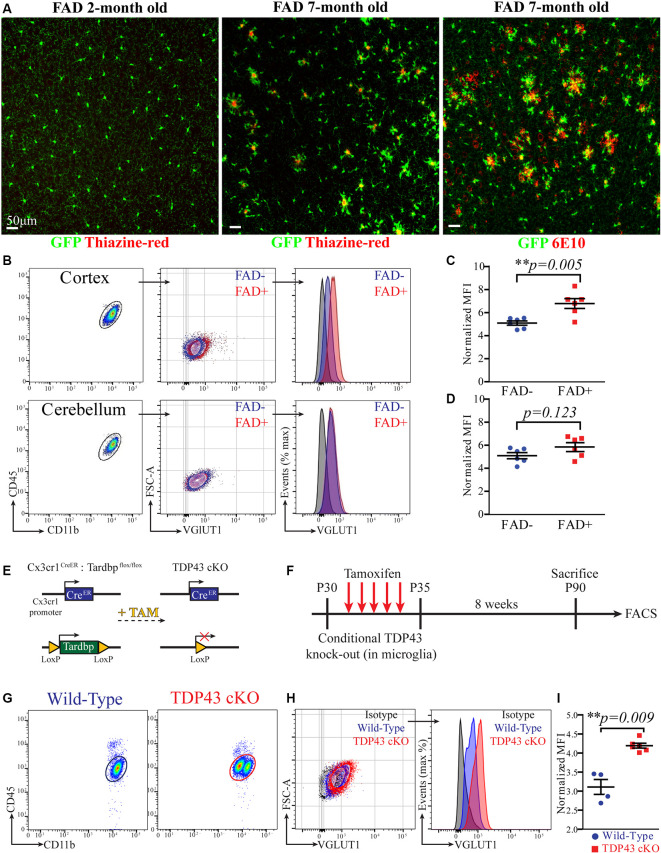
Microglia exhibit increased VGLUT1 immunoreactivity at early stages of amyloid pathology and after conditional TDP-43 deletion. **(A)** Confocal imaging in the cortex of 2-month-old and 7-month-old *5xFAD × Cx3cr1GFP/+* mice stained with either thiazine-red or the 6E10 antibody. Representative of three mice per group. **(B)** FACS plot of microglia extracted from either cortex or cerebellum. A comparison of the VGLUT1 IR between FAD+ and FAD− mice is displayed. **(C)** VGLUT1 n-MFI in cortical microglia from either FAD− or FAD+ mice (*N* = 6 mice per group; pooled data from two independent experiments; unpaired *t*-test; **p = 0.005). **(D)** VGLUT1 n-MFI in cerebellar microglia from either FAD− or FAD+ mice (*N* = 6 mice per group; pooled data from two independent experiments; unpaired *t*-test; non-significant difference). **(E)** Scheme of the conditional *Tardbp* knock-out in microglia. **(F)** Scheme of the experimental design. **(G)** FACS plot displaying microglia from wild-type and TDP-43 cKO mice. Note that TDP-43 cKO microglia appeared as two distinct populations. **(H)** VGLUT1 IR in microglia from wild-type and TDP-43 cKO mice. **(I)** VGLUT1 nMFI in TDP-43 cKO microglia compared to wild-type microglia (wild-type *N* = 4 mice, TDP-43 cKO *N* = 6 mice, data from a single experiment; Mann–Whitney *U*-test; **p = 0.009).

As main pathological hallmarks, Alzheimer’s disease (AD) is characterized by extracellular deposition of amyloid-β and intracellular accumulation of phospho-Tau. Alongside, AD brains feature a progressive loss of neurons and synapses (Ziegler-Waldkirch and Meyer-Luehmann, 2018). Interestingly, an increase of microglia-mediated synaptic pruning was recently indicated as an early pathophysiological event in a mouse model of AD before full-blown amyloid pathology (Hong et al., 2016). We then hypothesized that microglia may exhibit increased content of VGLUT1 protein at early stages of amyloid-β (Aβ) deposition. To test this hypothesis, we compared VGLUT1 IR in microglia from a mouse model of AD and wild-type controls. For this experiment, we decided to rely on the *5xTgFAD* (hereafter FAD+) mouse line crossed with the *Cx3cr1^GFP/+^* line to help visualize microglia during amyloid pathology. In mice carrying the FAD mutations, amyloid plaques typically start to develop in the subiculum at 2 months and in the hippocampus 4 months after birth. Nonetheless, intraneuronal Aβ_42_ and synaptic degeneration can be observed at earlier time points (Oakley et al., 2006). Importantly, amyloid pathology does not involve the cerebellum. First, we assessed the presence of the amyloid plaques in the cortex of 2 months old and 7 months old FAD+ mice using both the 6E10 antibody (staining for human APP) and the thiazine-red staining (staining for fibrillar Aβ). Confocal imaging showed that, at 2 months of age, no obvious amyloid deposits can be observed. By contrast, several amyloid plaques surrounded by plaque-associated microglia are clearly visible at 7 months (Figure 3A). This data indicates that FAD+ mice at age of 2 months do not exhibit signs of obvious amyloid pathology. We then analyzed VGLUT1 IR in microglia from the cortex of 2 months old FAD+ and FAD− (wild-type) mice, while microglia from the cerebellum were used as a negative control (Figure 3B). Our flow cytometry analysis revealed a slight, but significant, increase of the VGLUT1 n-MFI in cortical microglia from FAD+ compared to FAD− mice (6.8 ± 0.4 and 5.1 ± 0.2, respectively; Figure 3C). By contrast, cerebellar microglia did not show any significant difference between FAD+ and FAD− mice (5.8 ± 0.4 and 5.1 ± 0.3, respectively; Figure 3D). In immunohistochemistry, we assessed the overall density of VGLUT1 synapses in both cortex (Supplementary Figure S4A) and cerebellum (Supplementary Figure S4B) of 2 months old FAD+ and FAD− mice. Our quantification revealed a mild loss of VGLUT1 synapses in the cortex of FAD+ mice as compared to wild-type controls (Supplementary Figure S4C). Although the observed difference did not reach the statistical significance (*p* = 0.2), we can cautiously suggest that there is a tendency towards a loss of glutamatergic synapses at the age of 2 months. As expected, no difference in density of VGLUT1 synapses was found in the cerebellum (Supplementary Figure S4). To further corroborate these data, we assessed the number of VGLUT1-positive events in the cortical non-myeloid cells (CX_3_CR1^−^CD11b^−^CD45^−^ population) using flow cytometry (Supplementary Figure S4E). Consistently with the previous observation, a significant reduction (−19% ± 0.04) of the VGLUT1-positive events was found in the cortex of 2 months old FAD+ mice in comparison to wild-type mice (Supplementary Figure S4F). In summary, FAD+ mice display a (mild) loss of cortical glutamatergic synapses before the formation of amyloid plaques. At the very same time, cortical microglia exhibit increased immunoreactivity for VGLUT1, suggesting increased engulfment of glutamatergic synapses at early stages of amyloid pathology.

### Microglia Exhibit Increased VGLUT1 Immunoreactivity After Conditional TDP-43 Deletion

Recent work described augmented phagocytosis in microglia lacking the *Tardbp* gene, encoding for the RNA-DNA binding-protein TDP-43. The depletion of TDP-43 in microglia caused increased phagocytosis of amyloid-β, but also promoted loss of VGLUT1 synapses, probably because of augmented synaptic pruning (Paolicelli et al., 2017). As an additional benchmark for our quantification method, we decided to compare VGLUT1 IR between TDP-43-deficient and wild-type microglia. Mice selectively lacking TDP-43 in microglia were generated by crossing *Cx3cr1^CreERT2^* mice with *Tardbp^flox/flox^* mice (Parkhurst et al., 2013; Paolicelli et al., 2017; Figure 3E). In principle, this conditional Tamoxifen-inducible mouse model allows to specifically delete loxP-flanked genes in parenchymal microglia and other long-lived CNS-associated macrophages (Goldmann et al., 2013, 2016; Wieghofer et al., 2015). To further explore the presence of VGLUT1 protein in highly phagocytic microglia, we assessed the VGLUT1 IR in both *Cx3cr1^CreERT2^;Tardbp^+/+^* and *Cx3cr1^CreERT2^;Tardbp^flox/flox^* littermates (hereafter wild-type and TDP-43 cKO, respectively). Starting at P30, all mice underwent daily injections of Tamoxifen on five consecutive days to induce Cre-mediated recombination. Successful ablation of TDP-43 in microglia from *Tardbp^flox/flox^* carriers was previously demonstrated (Paolicelli et al., 2017). After 8 weeks, mice were euthanized, and brains were analyzed by flow cytometry (experimental plan in Figure 3F). Unlike wild-type mice, in the TDP-43 cKO group, we could observe the presence of two distinct sub-populations of microglia differing in CD11b intensity (Figure 3G). To explain this phenomenon, we hypothesize that some microglial cells failed to recombine both *Tardbp* alleles, thus generating two populations with different CD11b expression level. Whether or not this difference in CD11b expression is caused by an incomplete recombination of the *Tardbp^flox/flox^* locus remains to be established. Besides this unexpected finding, TDP-43 cKO microglia exhibited a remarkable increase of VGLUT1 IR (Figure 3H). Our quantification showed a significant increase of VGLUT1 n-MFI in TDP-43 cKO microglia as compared to wild-type (4.2 ± 0.1 and 3.1 ± 0.2, respectively; Figure 3I). Again, VGLUT1 IR was not detectable in CD45^hi^ MPs (not shown). These data support previous evidence that TDP-43 ablation in microglia leads to increased engulfment of glutamatergic synapses (Paolicelli et al., 2017).

## Discussion

The first evidence of microglia displacing synapses from neuronal cell bodies was presented approximately 50 years ago (Blinzinger and Kreutzberg, 1968). Over the last decade, the scientific community has witnessed a growing interest in this topic (Tremblay et al., 2010; Paolicelli et al., 2011; Schafer et al., 2012). In the mouse brain, the first 2 weeks of postnatal development are accompanied by a peak in synaptic turnover, with intense *de novo* synapse formation and synapse elimination. Microglia critically contribute to the removal of exuberant and unnecessary synapses generated across this period, playing an important role in synaptic pruning. To date, the molecular cues implicated in this process are largely unknown and it is possible that different brain cells, other than microglia, secrete factors modulating this process (Bialas and Stevens, 2013; Vainchtein et al., 2018). It has been so far shown that a microglia-mediated synaptic pruning occurs during brain development, while evidence of synaptic pruning in the healthy adult brain is still lacking.

We first sought evidence of colocalization between the pre-synaptic marker VGLUT1 and microglial cytoplasm in the hippocampus of adult mice by confocal imaging. Our confocal and STED images disclosed the presence of the pre-synaptic protein VGLUT1 within microglial processes and cell bodies. Moreover, such VGLUT1 inclusions were often colocalized with the phagosomes, indicating that synaptic pruning may still occur in the hippocampus during adulthood. To provide a quantitative appraisal of the VGLUT1 protein in microglia we assessed VGLUT1 immunoreactivity in the CD11b^+^CD45^lo^ population by flow cytometry. Our data show that microglia in the adult brain contain a detectable amount of VGLUT1 protein, which can be successfully stained and quantified *via* fluorescence intensity. Using this approach, we could also show that microglia from the developing brain contain more than two times the amount of VGLUT1 found in adult microglia.

Next, we aimed to quantify VGLUT1 in microglia from two pathological models. First, we assessed the VGLUT1 staining in microglia from the 5xFAD mouse model of AD. We found that cortical microglia exhibit a significant increase of immunoreactivity for VGLUT1 in 2 months old 5xFAD mice as compared to wild-types. This suggests that microglia may account, at least partially, for the synaptic degeneration observed in the early stages of the pathology (Hong et al., 2016; Shi et al., 2017). Microglia may remove injured/dysfunctional glutamatergic synapses at early stages of amyloid pathology *via* synaptic pruning. Alternatively, we may hypothesize that Aβ-related toxicity causes a loss of glutamatergic synapses, debris of which is rapidly scavenged by neighboring microglia. These two scenarios are not mutually exclusive, and further studies are needed to determine whether microglia are directly involved in the synaptic loss observed under AD-like pathology.

Last, we assessed the presence of VGLUT1 protein in microglia from the *Cx3cr1^CreERT2^;Tardbp^flox/flox^* mouse line. As recently described, conditional TDP-43 deletion in microglia (TDP-43 cKO) induces a highly phagocytic phenotype, which develops alongside the loss of VGLUT1 synapses from the cortical parenchyma (Paolicelli et al., 2017). In flow cytometry, we could detect a significant increase in VGLUT1 immunoreactivity in TDP-43 cKO microglia, suggesting augmented pruning of glutamatergic synapses. This evidence further corroborates (and integrates) the previously published data, indicating that the synaptic loss observed in this transgenic mouse is likely contributed by exaggerated synaptic pruning.

The quantification of synaptic proteins in microglia by flow cytometry certainly provides some advantages compared to the traditional microscopy techniques, such as a much faster acquisition and analysis of the experimental data. Nevertheless, there are some important caveats to keep in mind: (1) Application of this technique is limited by the availability of antibodies specific for synapses and, at the same time, suitable for flow cytometry staining. (2) Flow cytometry requires tissue homogenization with loss of the structural and anatomical information of the brain’s parenchyma, that is otherwise preserved in histology. (3) Using flow cytometry, we are unable to provide a direct visualization of the VGLUT1 immunoreactivity in microglia, which would help determine the exact localization of the VGLUT1 inclusions. (4) The presented data do not provide experimental proof that microglia actively prune synapses from the neuronal dendrites as opposed to “simply” scavenging synaptic debris within the brain parenchyma. (5) This method is not specific for synaptic proteins; indeed, we could also detect immunoreactivity for GFAP, MAP2, and NeuN in microglia (not shown), possibly owing to the uptake of dead cells in the brain.

It is important to note that, in this study, VGLUT1 immunoreactivity was never found in CD45^hi^ (extra-parenchymal) macrophages or splenic macrophages, indicating that the staining did not produce non-specific labeling of myeloid antigens. Moreover, we provided evidence that the observed VGLUT1 immunoreactivity was intracellular and not merely due to adhesion of VGLUT1 proteins to the cell surface.

In conclusion, with this study, we aimed to perform relative quantification of the synaptic protein VGLUT1 in microglia during brain development, adulthood, and neurodegeneration using flow cytometry. This work corroborates previous evidence, mostly based on imaging techniques, providing a methodological framework for quantitative assessment which may help investigate microglia-mediated synapse elimination. A better understanding of the pathological synaptic pruning will help design novel therapies for neurodevelopmental and neurodegenerative disorders.

## Data Availability Statement

All datasets presented in this study are included in the article/Supplementary Material.

## Ethics Statement

The animal study was reviewed and approved by committee of the University of Freiburg/Swiss Cantonal Veterinary Office.

## Author Contributions

SB designed the experiments, performed the experiments and wrote the manuscript. PD’E, LA, HJ, PW and RP performed the experiments. SW, MM-L, LR and KB provided animals and/or materials. All authors edited the manuscript. PW, RP and KB supervised the project.

## Conflict of Interest

The authors declare that the research was conducted in the absence of any commercial or financial relationships that could be construed as a potential conflict of interest.

## Footnotes

^1^https://svi.nl

^2^https://www.bitplane.com

^3^https://www.abberior-instruments.com

## Supplementary Material

FIGURE S1 **(A)** Confocal image in the hippocampus of a Thy.1-eGFP mouse with anti-Iba1 staining showing the interactions between microglial processes and neuronal dendrites (white arrowheads). A GFP^+^ inclusion within the microglial cytoplasm is also visible (yellow arrowhead). **(B)** Immunohistochemistry in the mouse cortex for Iba1 (green) and VGLUT1 (red). Diffuse VGLUT1 staining is present in wild-type (*Vglut1^+/+^*), but not in the knock-out (*Vglut1*^−/−^) brain. **(C)** VGLUT1 synapses (red) were found in proximity to Thy1-GFP neurons (green). 3D reconstruction highlights the close contact between VGLUT1 synapses and the neuronal dendrites. **(D)** A microglial cell in the CA1 exhibiting a VGLUT1 inclusion within a process (white box and yellow arrowhead). **(E)** A microglial cell in the CA1 exhibiting VGLUT1 inclusions in the cell body (white box and yellow arrowhead).

FIGURE S2 Gating strategy for microglia, brain CD45^hi^ MPs and splenic MPs. Cells were hierarchically gated as follows: CD11b^+^ brain’s myeloid cells → single cells → viable cells → GFP^+^ or GFP^−^ (depending on the presence of the *Cx3cr1^GFP/+^* transgene) → CD11b^+^CD45^lo^ population (microglia) and CD11b^+^CD45^hi^ population (CD45^hi^ MPs). Representative gating strategy for **(A)** wild-type brain, **(B)** *Cx3cr1^GFP/+^* brain, and **(C)** spleen.

FIGURE S3 **(A)** VGLUT1 immunoreactivity is present in the brain tissue, but not in the spleen. **(B)** VGLUT1 n-MFI in microglia from either cortex or hippocampus (*N* = 6 mice per group; pooled data from two independent experiments; unpaired *t*-test; **p* = 0.0149). **(C)** FACS plot showing all brain cells from *Cx3cr1^GFP/+^* mice, gated as CD11b^+^ and CD11b^−^ populations. **(D)** Microglia were gated as CD11b^+^CX_3_CR1^+^CD45^lo^ population. **(E)** Non-myeloid cells were gated as CD11b^−^CX_3_CR1^−^CD45^−^ population. VGLUT1 IR in either gate was detectable only in presence of permeabilization buffer. **(F)** VGLUT1 n-MFI in microglia stained with either FACS buffer or permeabilization buffer (*N* = 4 samples per group; data from a single experiment; Mann–Whitney *U*-test; **p* < 0.028). **(G)** VGLUT1 MFI fold-change between non-permeabilized and permeabilized microglia (four samples per group).

FIGURE S4 **(A)** Iba1 (red) and VGLUT1 (green) staining in the cortex of FAD− and FAD+ mice at the age of 2 months. **(B)** Iba1 (red) and VGLUT1 (green) staining in the cerebellum of FAD− and FAD+ mice at the age of 2 months. **(C)** Quantification of VGLUT1+ puncta per field (100 μm^2^) in the cortex of FAD− and FAD+ mice at 2 months (*N* = 3 mice per group, average of two sections per mouse; data from a single experiment; Mann–Whitney *U*-test; non-significant difference). **(D)** Quantification of VGLUT1+ puncta per field (100 μm^2^) in the cerebellum of FAD− and FAD+ mice at 2 months (*N* = 3 mice per group, average of two sections per mouse; data from a single experiment; Mann–Whitney *U*-test; non-significant difference). **(E)** VGLUT1 immunoreactivity in the non-myeloid cells (CD11b−CD45−) from the cortex of either FAD− and FAD+ mice at 2 months. **(F)** Percentage of the VGLUT1-positive events in the brain’s non-myeloid cells (gated as in panel **E**) were measured. The plot displays the fold change in the FAD+ group (*N* = 4 mice per group; data from a single experiment; Mann–Whitney *U*-test; **p* < 0.029).
