# Supplementary figures and images for: Detection of Synaptic Proteins in Microglia by Flow Cytometry
Source: Front Mol Neurosci. 2020 Sep 29;13:149. doi: 10.3389/fnmol.2020.00149 (PMC7550663; doi:10.3389/fnmol.2020.00149)

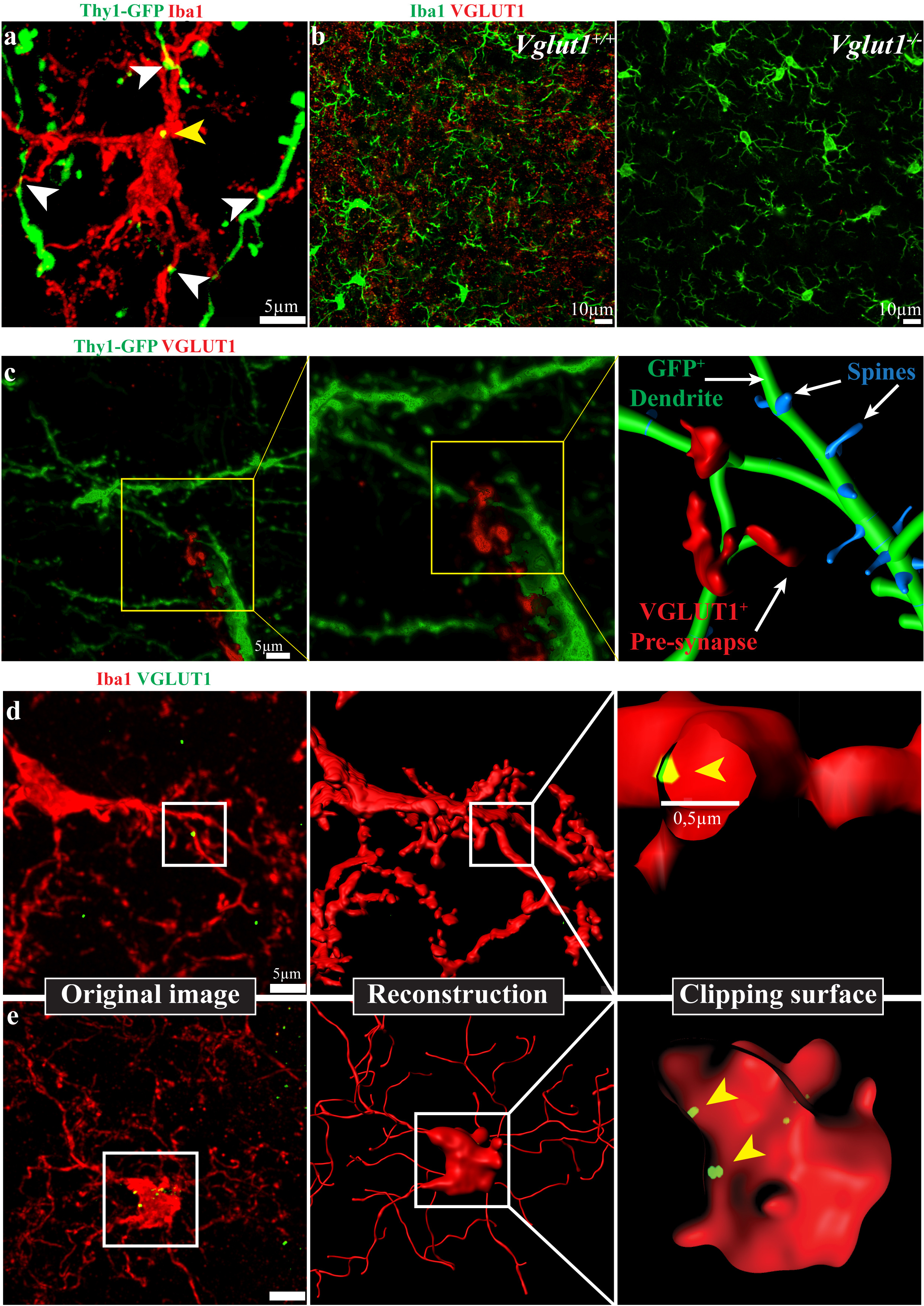

Supplement: FIGURE S1 — (A) Confocal image in the hippocampus of a Thy.1-eGFP mouse with anti-Iba1 staining showing the interactions between microglial processes and neuronal dendrites (white arrowheads). A GFP+ inclusion within the microglial cytoplasm is also visible (yellow arrowhead). (B) Immunohistochemistry in the mouse cortex for Iba1 (green) and VGLUT1 (red). Diffuse VGLUT1 staining is present in wild-type (Vglut1+/+), but not in the knock-out (Vglut1−/−) brain. (C) VGLUT1 synapses (red) were found in proximity to Thy1-GFP neurons (green). 3D reconstruction highlights the close contact between VGLUT1 synapses and the neuronal dendrites. (D) A microglial cell in the CA1 exhibiting a VGLUT1 inclusion within a process (white box and yellow arrowhead). (E) A microglial cell in the CA1 exhibiting VGLUT1 inclusions in the cell body (white box and yellow arrowhead). [file Image_1.jpeg]

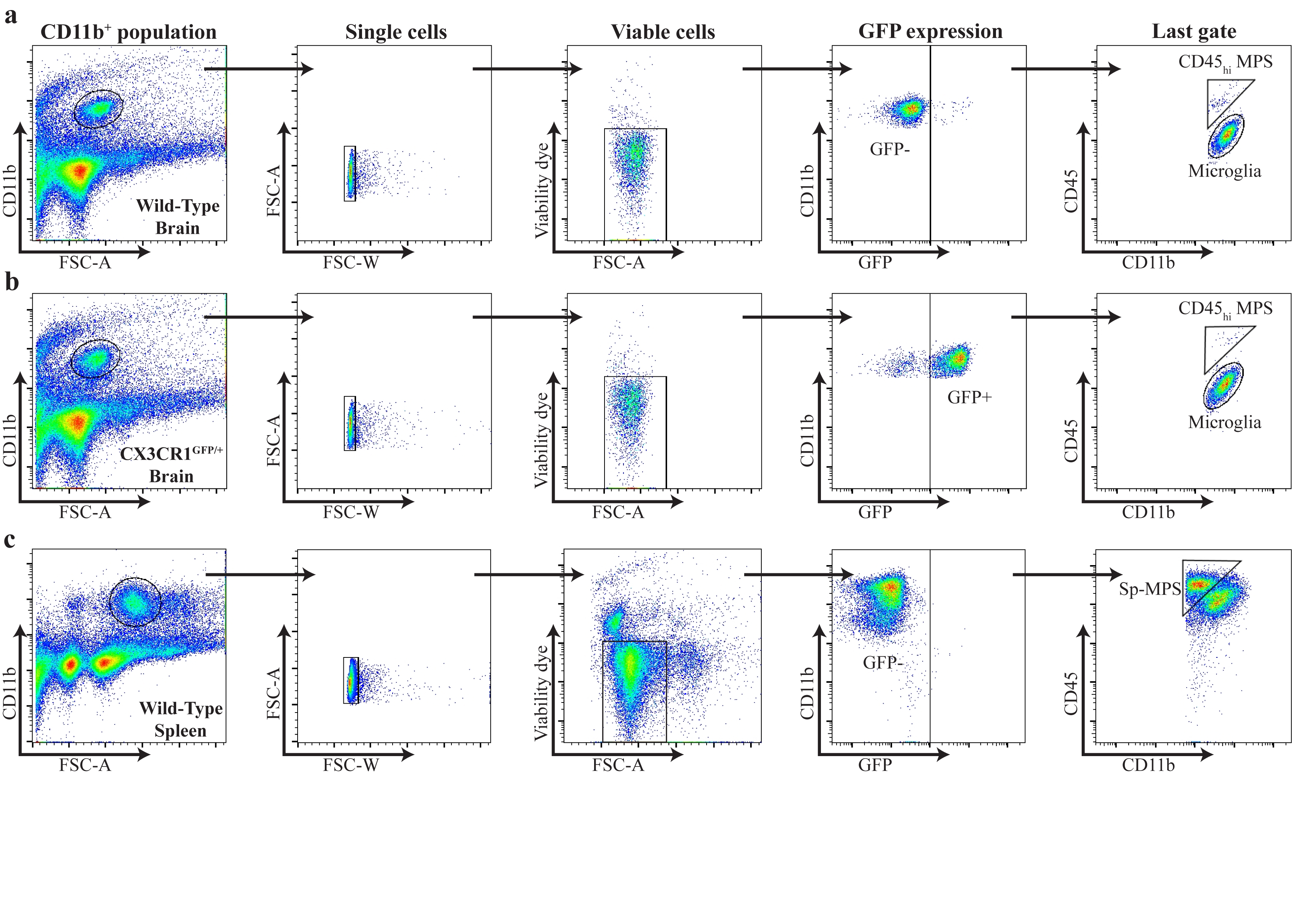

Supplement: FIGURE S2 — Gating strategy for microglia, brain CD45hi MPs and splenic MPs. Cells were hierarchically gated as follows: CD11b+ brain’s myeloid cells → single cells → viable cells → GFP+ or GFP− (depending on the presence of the Cx3cr1GFP/+ transgene) → CD11b+CD45lo population (microglia) and CD11b+CD45hi population (CD45hi MPs). Representative gating strategy for (A) wild-type brain, (B) Cx3cr1GFP/+ brain, and (C) spleen. [file Image_2.jpeg]

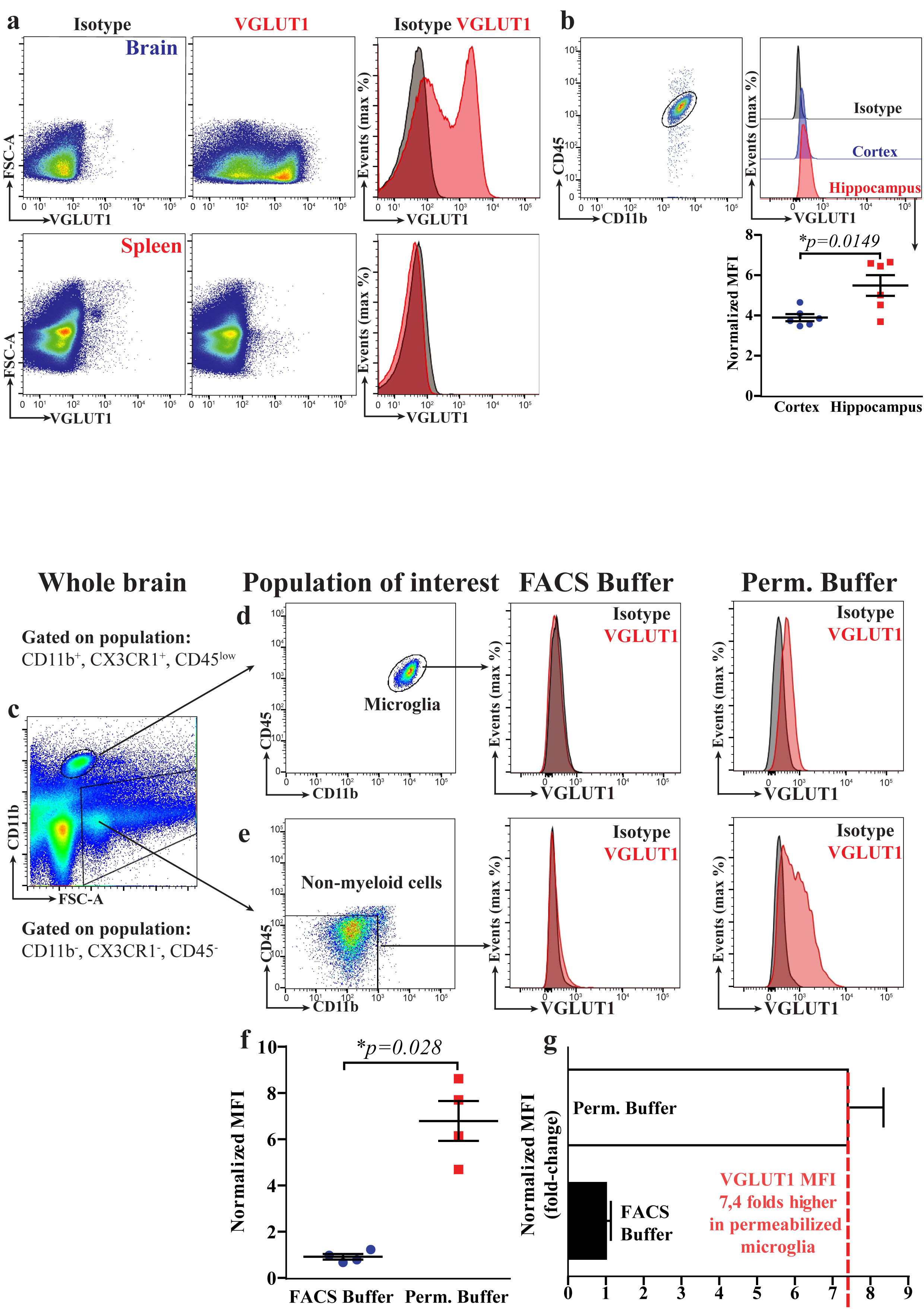

Supplement: FIGURE S3 — (A) VGLUT1 immunoreactivity is present in the brain tissue, but not in the spleen. (B) VGLUT1 n-MFI in microglia from either cortex or hippocampus (N = 6 mice per group; pooled data from two independent experiments; unpaired t-test; *p = 0.0149). (C) FACS plot showing all brain cells from Cx3cr1GFP/+ mice, gated as CD11b+ and CD11b− populations. (D) Microglia were gated as CD11b+CX3CR1+CD45lo population. (E) Non-myeloid cells were gated as CD11b−CX3CR1−CD45− population. VGLUT1 IR in either gate was detectable only in presence of permeabilization buffer. (F) VGLUT1 n-MFI in microglia stained with either FACS buffer or permeabilization buffer (N = 4 samples per group; data from a single experiment; Mann–Whitney U-test; *p < 0.028). (G) VGLUT1 MFI fold-change between non-permeabilized and permeabilized microglia (four samples per group). [file Image_3.jpeg]

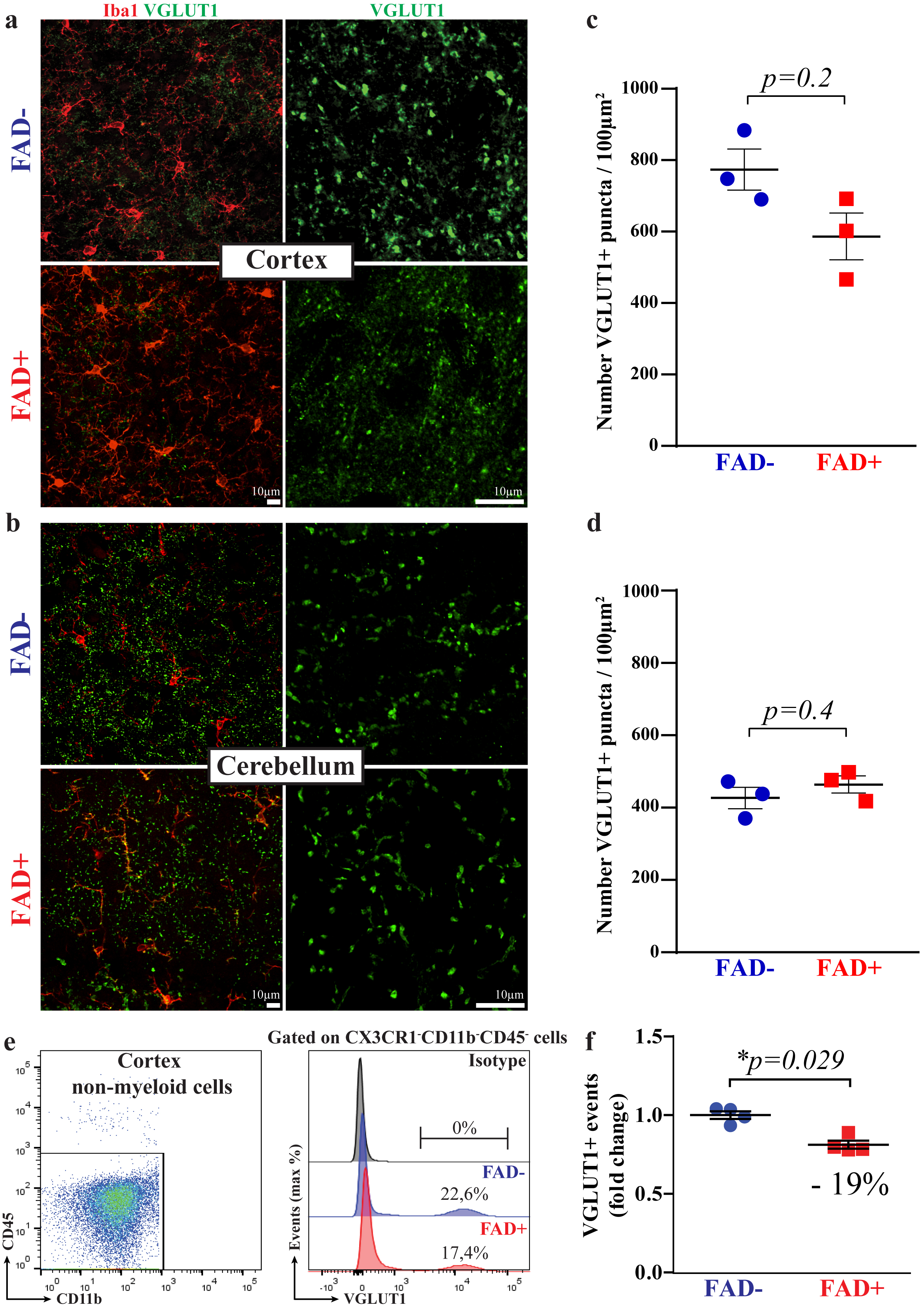

Supplement: FIGURE S4 — (A) Iba1 (red) and VGLUT1 (green) staining in the cortex of FAD− and FAD+ mice at the age of 2 months. (B) Iba1 (red) and VGLUT1 (green) staining in the cerebellum of FAD− and FAD+ mice at the age of 2 months. (C) Quantification of VGLUT1+ puncta per field (100 μm2) in the cortex of FAD− and FAD+ mice at 2 months (N = 3 mice per group, average of two sections per mouse; data from a single experiment; Mann–Whitney U-test; non-significant difference). (D) Quantification of VGLUT1+ puncta per field (100 μm2) in the cerebellum of FAD− and FAD+ mice at 2 months (N = 3 mice per group, average of two sections per mouse; data from a single experiment; Mann–Whitney U-test; non-significant difference). (E) VGLUT1 immunoreactivity in the non-myeloid cells (CD11b−CD45−) from the cortex of either FAD− and FAD+ mice at 2 months. (F) Percentage of the VGLUT1-positive events in the brain’s non-myeloid cells (gated as in panel E) were measured. The plot displays the fold change in the FAD+ group (N = 4 mice per group; data from a single experiment; Mann–Whitney U-test; *p < 0.029). [file Image_4.jpeg]
