# Supplementary material for: Detection of Synaptic Proteins in Microglia by Flow Cytometry
Source: Front Mol Neurosci. 2020 Sep 29;13:149. doi: 10.3389/fnmol.2020.00149 (PMC7550663; doi:10.3389/fnmol.2020.00149)
Supplement: Supplementary file 5 [file Data_Sheet_1.docx]

**FACS intracellular staining protocol**

1) Myelin removal protocol

- Perfuse the mouse and mash the brain with the tissue potter
- Filter the brain homogenate through a 70um strainer with 50ml of HBSS media
- Centrifuge at 200g/10 min at 4°C
- Discard the supernatant and re-suspend the pellet in 5ml of Percoll 35%, then overlay the percoll fraction with 2ml of PBS
- Centrifuge at 1000g/30 min at 4°C (acceleration 4, break 0)
- Myelin will settle at the interface between Percoll and PBS. Remove the myelin by aspiration with a Pasteur pipette. Then discard all the supernatant by tube inversion. The pellet contains all brain cells (including microglia).
- Re-suspend the pellet in 10ml of PBS (pellet wash) and centrifuge again at 200g/10 min at 4°C. In the end discard the supernatant.

**Note:** Splenocytes were collected by squeezing chopped spleen tissue on a 70µm strainer filter, subsequently rinsed with 50ml of PBS. Cellular fraction was collected into a 50ml falcon tube and centrifuged. Pellet was re-suspended in 1ml of red blood cells lysis buffer (Sigma) and eventually washed in 10ml PBS. After centrifugation, splenocytes pellet was transferred into FACS tubes.

2) FACS staining protocol

- Re-suspend well pellet in 500ul PBS (no FACS buffer) and transfer the pellet in a FACS tube. Centrifuge at 300g/5 min at 4°C
- Discard supernatant and add 100ul/sample of live/dead staining solution (eBioscience Fixable Viability Dye eFlur780, dilution 1:1000 in PBS, 5 min on ice). Vortex for about 5-10 seconds for re-suspending the cells attached on the bottom of the tube.
- Fill the tube with PBS and centrifuge as before
- Discard supernatant and add Fc-blocker (eBioscience anti-mouse CD16/CD32 Monoclonal Antibody, dilution 1:200 in FACS buffer, 10 min on ice). Vortex for about 5-10 seconds
- Fill the tube with FACS buffer and centrifuge as before
- Discard supernatant and add 100ul of surface labelling solution containing anti-Cd11b-BV421 (Biolegend, clone M1/70) and anti-CD45-APC (eBioscience, clone 30-F11). For both antibodies: dilution 1:200 in FACS buffer, 30 min in the fridge 4°C, keep at the dark). Vortex for about 5-10 seconds.
- Fill the tube with FACS buffer and centrifuge as before
- Discard supernatant and start the fixation/permeabilization step. Protocol adapted from BD Cytofix/Cytoperm.

**Note:** We used the Fixation/Permeabilization Solution Kit (BD Bioscience), but any other fixation/permeabilization buffer should work similarly.

3) Fixation/permeabilization protocol

- Thoroughly re-suspend cells with vortex and add 250μl of Fixation/Permeabilization solution (20 minutes on ice, at the dark).
- Wash cell suspension with 2ml of 1X Perm/Wash buffer (BD Bioscience provides a 10X stock solution). Centrifuge as before.
- Discard supernatant and repeat step #2 (wash twice). Note permeabilization is reversible, therefore cells must be maintained in Perm/Wash buffer during all the following steps to keep them permeabilized.
- Discard supernatant and start the intracellular staining step

4) Intracellular staining protocol

- Add 100ul of intracellular staining solution containing the PE-labelled anti-VGLUT1 antibody (0.5ug/test, diluted in Perm/Wash buffer). Isotype CTRL samples will contain the same concentration of IgG-PE Isotype-CTRL. We use the Anti-Mouse Vesicular Glutamate Transporter 1-PE Antibody (Milli-Mark, clone 3C10.2) and the Mouse IgG1 K Isotype Control PE (eBioscience, Clone P3.6.2.8.1).
- Vortex and incubate at 4°C overnight
- Wash cell suspension with 2ml of 1X Perm/Wash buffer and centrifuge as before. Discard supernatant and repeat the washing step (wash twice).
- Discard supernatant, fill the tube with FACS buffer and centrifuge as before. The last wash with FACS buffer allows removal of the permeabilization buffer (stop permeabilization).
- Re-suspend cells in 300ul of FACS buffer and flow through 40um strainer filter
- Read samples in flow cytometry.
